# Subcutaneous Adipose Fatty Acid Profiles and Related Rumen Bacterial Populations of Steers Fed Red Clover or Grass Hay Diets Containing Flax or Sunflower-Seed

**DOI:** 10.1371/journal.pone.0104167

**Published:** 2014-08-05

**Authors:** Renee M. Petri, Cletos Mapiye, Mike E. R. Dugan, Tim A. McAllister

**Affiliations:** 1 Lethbridge Research Centre, Agriculture and Agri-Food Canada, Lethbridge, Alberta, Canada; 2 Lacombe Research Centre, Agriculture and Agri-Food Canada, Lacombe, Alberta, Canada; 3 Department of Animal Sciences, Faculty of AgriSciences, Stellenbosch University, Matieland, Western Cape, South Africa; National Institute of Nutrition, India

## Abstract

Steers were fed 70∶30 forage∶concentrate diets for 205 days, with either grass hay (GH) or red clover silage (RC), and either sunflower-seed (SS) or flaxseed (FS), providing 5.4% oil in the diets. Compared to diets containing SS, FS diets had elevated (*P*<0.05) subcutaneous *trans* (*t*)-18:1 isomers, conjugated linoleic acids and *n*-6 polyunsaturated fatty acid (PUFA). Forage and oilseed type influenced total *n*-3 PUFA, especially α-linolenic acid (ALA) and total non-conjugated diene biohydrogenation (BH) in subcutaneous fat with proportions being greater (*P*<0.05) for FS or GH as compared to SS or RC. Of the 25 bacterial genera impacted by diet, 19 correlated with fatty acids (FA) profile. *Clostridium* were most abundant when levels of conjugated linolenic acids, and *n*-3 PUFA's were found to be the lowest in subcutaneous fat, suggestive of their role in BH. *Anerophaga, Fibrobacter, Guggenheimella, Paludibacter and Pseudozobellia* were more abundant in the rumen when the levels of VA in subcutaneous fat were low. This study clearly shows the impact of oilseeds and forage source on the deposition of subcutaneous FA in beef cattle. Significant correlations between rumen bacterial genera and the levels of specific FA in subcutaneous fat maybe indicative of their role in determining the FA profile of adipose tissue. However, despite numerous correlations, the dynamics of rumen bacteria in the BH of unsaturated fatty acid and synthesis of PUFA and FA tissue profiles require further experimentation to determine if these correlations are consistent over a range of diets of differing composition. Present results demonstrate that in order to achieve targeted FA profiles in beef, a multifactorial approach will be required that takes into consideration not only the PUFA profile of the diet, but also the non-oil fraction of the diet, type and level of feed processing, and the role of rumen microbes in the BH of unsaturated fatty acid.

## Introduction

The healthfulness of beef has been challenged because of its relatively high concentrations of saturated fatty acids (SFA [1]), including myristic (14:0) and palmitic (16:0) acids which have been shown to raise serum levels of low-density lipoproteins, a risk factor for cardiovascular disease in humans [2]. However, meat also contains essential fatty acids (EFA) such as α-linolenic acid (18:3*n*-3, ALA) and its elongation and desaturation products including eicosapentaenoic acid (20:5*n*-3, EPA), docosapentenoic acid (22:5*n*-3) and docosahexaenoic acid (22:6*n*-3; DHA) and rumen biohydrogenation (BH) products including rumenic acid (*c*9,*t*11-18:2, RA) and its precursor vaccenic acid (*t*11-18:1, VA) which have purported human health-promoting properties [3]–[6]. In this context, current research efforts have been directed at finding dietary strategies that facilitate higher fore-stomach bypass of *n*-3 polyunsaturated fatty acids (PUFA) and specific PUFA BH products (*i.e*, VA and RA) for absorption and incorporation into adipose tissue.

Our previous studies have evaluated the effects of feeding 10 to 15% flaxseed (FS, a rich source of ALA) in the diet on ALA and its BH products in beef [7]–[9]. In an initial examination, feeding steers FS in a barley grain-based (73% of dry matter [DM]) diet resulted in limited absolute increases *n*-3 PUFA and their BH products in beef. In this research, the BH pathway promoted tissue accumulation of *t*13-/*t*14-18:1 rather than VA, and non-conjugated diene BH products (*i.e.*, atypical dienes, AD) in place of conjugated linoleic acids (CLA) [8]. In a second investigation, we fed cull cows grass hay (GH) or barley silage-based (50% of DM) diets supplemented with FS for 20 weeks and found that cows fed GH had a greater concentration of *n*-3 PUFA and BH products, with VA as the main *t*-18:1 isomer, as well as greater levels of AD instead of CLA in adipose tissue as compared to those fed barley silage [7], [9].

Recent research in our laboratory has shown that supplementing a red clover silage (RC, 70% of DM) diet with FS for 215 days resulted in greater levels of *n*-3 PUFA and its BH products as a proportion of total fatty acids (FA) when compared to feeding other silages. As much as 2.9% of RA was found in the subcutaneous fat of cattle fed a RC diet supplemented with FS [10]. In this research, the increased amounts of PUFA BH products were in part associated with the amount and duration of FS feeding and the presence of relatively increased levels of polyphenol oxidase (PPO) activity which can produce quinones in RC, reducing the rate of PUFA lipolysis and BH in the rumen [11]. Consequently, adipose deposition of PUFA and their BH products was increased [12]. To our knowledge, no studies have directly compared RC with other forages for its effect on the concentrations of PUFA and BH intermediaries in beef adipose tissue [7].

Results from comparisons of different oilseeds suggest that those rich in linoleic acid (LA, 18:2*n*-6) such as sunflower-seed (SS) may be more effective at increasing VA and CLA in beef [13]–[15], while those rich in ALA increase ALA and its specific BH products. In this regard, it may be important to investigate how the deposition of VA, CLA and *n*-3 PUFA in beef differs when feeding RC as opposed to GH supplemented with SS or FS as PUFA sources.

Previously we have observed large coefficients of variation in concentrations of PUFA BH products among cattle, especially for VA, when feeding FS in high-forage diets [7], [16], but the source of this inter-animal variation remains uncertain. Overall, differences in FA composition have been reported to originate from inter-animal variation in the rumen environment, including the kinds and numbers of rumen microbes present as well as other factors [17]. In this respect, determining an individual animal's rumen microbial profile with current metagenomic technologies could be useful in minimizing FA variation amongst animals consuming the same diet by designing management strategies that enhance the levels of beneficial FA in beef through microbial manipulation. It may also help to identify those microbial populations involved in BH that are inhibited by plant secondary compounds such as the quinones in RC, an outcome that results in a more favorable FA profile in beef. The objectives of the current research were to compare the effects on rumen bacterial populations and related subcutaneous FA profiles when steers were fed GH or RC diets supplemented with FS or SS. Subcutaneous fat was chosen as a representative tissue as it is used to make hamburger, which is the most consumed beef product in North America, and it is considered a more representative indicator of rumen FA metabolism than muscle [18].

## Materials and Methods

### Animals and diets

Animal management and diets were previously described by Mapiye et al. [19] with ethical experimental practices reviewed and approved (Protocol #201102) by Lacombe Research Centre Animal Care Committee using guidelines which are accredited by the national Canadian Council of Animal Care [20]. Briefly, 64 British × Continental crossbred steers were stratified by weight to four experimental diets, with two pens of eight steers per diet. The four diets were GH-FS, GH-SS, RC-FS and RC-SS. On a dry matter (DM) basis, diets contained 70% forage and 30% concentrate with sunflower-seed (SS) or flaxseed (FS) at a level that resulted in the addition of 5.4% oil to the diets (Table 1). In an attempt to equalize the digestible energy of the diets, additional ground-barley grain was also included in diets containing SS, and additional barley straw was added to diets containing FS. Flaxseed was triple rolled, while SS was fed whole. Nutrient and FA composition of the experimental diets are also shown in Table 1.

**Table 1:** Ingredient, nutrient and fatty acid composition of the experimental diets.

	Diet^1^			
Variable	GH-FS	GH-SS	RC-FS	RC-SS
*Ingredient (% DM basis)*				
Red clover silage	0.0	0.0	70.0	70.0
Grass hay	70.0	70.0	0.0	0.0
Barley straw	11.5	0.0	11.5	0.0
Sunflower-seed	0.0	18.4	0.0	18.4
Flaxseed	14.3	0.0	14.3	0.0
Vitamin/mineral supplement^2^	4.2	4.2	4.2	4.2
Barley grain	0.0	7.4	0.0	7.4
*Nutrient (DM basis)*				
Dry matter (%)	93.1	93.0	46.9	46.9
Crude protein (%)	13.3	13.4	14.2	14.0
Crude fat (%)	6.4	6.6	8.2	8.4
Calcium (%)	1.1	1.1	1.1	1.2
Phosphorus (%)	0.3	0.3	0.3	0.2
ADF (%)	44.3	45.4	43.0	44.0
NDF (%)	53.2	57.6	55.5	61.6
Digestible Energy^3^ (Mcal/kg)	2.08	2.02	2.16	2.10
*Fatty acid (% of total fatty acids)*				
14:0	0.2	0.2	0.1	0.1
16:0	8.6	10.2	7.5	8.4
18:0	3.0	4.1	2.9	4.2
20:0	0.4	0.5	0.3	0.4
22:0	0.7	0.9	0.4	0.8
24:0	0.6	0.5	0.4	0.4
*c*9-18:1	11.6	11.3	11.6	11.7
*c*11-18:1	0.8	0.9	0.8	0.7
18:2*n*-6	23.4	66.0	21.4	70.4
18:3*n*-3	50.7	5.3	54.6	2.8

1 GH-FS, grass hay + flaxseed; GH-SS, grass hay + sunflower-seed, RC-FS, red clover silage + flaxseed; RC-SS, red clover silage + sunflower-seed.

2 Vitamin/mineral supplement per kg DM contained 1.86% calcium, 0.93% phosphorous, 0.56% potassium, 0.21% sulphur, 0.33% magnesium 0.92% sodium, 265 ppm iron, 314 ppm manganese, 156 ppm copper, 517 ppm zinc, 10.05 ppm iodine, 5.04 ppm cobalt, 2.98 ppm selenium, 49722 IU/kg vitamin A, 9944 IU/kg vitamin D3, and 3222 IU/kg vitamin E.

3 Digestible energy was calculated according to Bull, [72].

### Sample collection procedures

Subcutaneous fat thickness was measured monthly by a certified ultrasound technician using an Aloka 500V diagnostic real-time ultrasound with a 17 cm 3.5 Mhz linear array transducer (Overseas Monitor Corporation Ltd., Richmond, B.C., Canada) following procedures of Brethour, [21]. Steers were slaughtered at the Lacombe Research Centre abattoir over four slaughter dates in November 2011 (two steers/pen/diet/slaughter day) at an average of 205 d on feed corresponding to subcutaneous fat depths of 6–8 mm between the 12^th^ and 13^th^ rib over the right *longissimus thoracis* muscle of each animal.

On the morning of slaughter, steers were transported 2 km to the Lacombe Research Centre abbatoir for immediate slaughter. At slaughter, final live weights were recorded and steers were stunned, exsanguinated and dressed in a commercial manner. At approximately 20 min *post-mortem*, samples of subcutaneous fat adjacent to the 12^th^ rib were collected and stored at −80°C until analysed for FA. About 30 min *post-mortem*, the rumen was opened and the ruminal contents collected and thoroughly mixed. Thereafter, samples of ruminal contents (solids and fluid) were taken from mid-ventral region of the rumen (250 g) by the same researcher throughout the trial and placed into an open 2 L plastic container. Samples were then hand-mixed, subsampled and put in 2×50 ml plastic culture tubes. The tubes were immediately flash-frozen in liquid nitrogen and stored in a −80 freezer until DNA was extracted.

### Bacterial DNA extraction, sequencing and quantification

From the 64 steers, 24 were selected for rumen bacterial analysis, based on which six animals in each dietary treatment had the lowest (n = 3) or the highest (n = 3) vaccenic acid in their subcutaneous fat. Subsamples of ruminal contents (25–40 ml) were lyophilized in a VirTis Freezemobile 25 freeze dryer (SP Industries Warminste, PA, USA), and 2–4 g of dried material was ground for 5 minutes at a frequency of 30 cycles/s in 10 ml grinding jars with a 20 mm stainless steel ball using a Qiagen TissueLyser II (Qiagen, Toronto, ON). Total DNA was extracted from dried, ground samples (30 mg) in two parallel procedures: one from the initial lysis supernatant, and the other from the initial lysis pelleted fraction of the QIAamp DNA Stool Kit as described by Narvaez et al. [22]. Final DNA elution volume was 150 µl for the supernatant fraction, and 100 µl for the pelleted fraction. Concentrations of DNA were determined spectrophotometrically using a NanoDrop 2000 (ThermoScientific, Wilmington, DE, USA), and supernatant and pellet elutions were pooled 1∶1 (v/v).

Bacterial tag-encoded FLX-Titanium amplicon pyrosequencing (bTEFAP) of the pooled DNA, [23], was performed at MR DNA (Shallowater, TX, USA). Bacterial 16S primers 530F 5′–GTGCCAGCMGCNGCGG-3′ and 1100R 5′-GGGTTNCGNTCGTTG-3′ were used in PCR amplification of the V4–V6 hyper-variable regions of 16S rRNA gene. Sequencing primers and barcodes were trimmed from the DNA sequences and quality control measures using Mothur [24] were used to exclude sequences <200 bp or those containing homopolymers longer than 8 base pairs. Pyrosequencing errors were minimized in the dataset, using the pre-cluster algorithm in Mothur [25], whereby rare sequences highly similar to abundant sequences were re-classified as their abundant homologue. Chimeras were removed from the samples, using the sequence collection as its own reference database [24]. Clean reads were submitted to EBI European Nucleotide Archive (ENA) database (http://www.ebi.ac.uk/ena, accession number PRJEB6402). Calculation of treatment based rarefaction curves, using the Mothur pipeline provided a way of comparing the phylogenetic richness among samples and determining the extent of sequencing relative to sampling needed to accurately describe the microbial community. While the total number of sequences obtained was decreased in the RC-FS compared to the other diets, the degree of coverage was similar across diets (Fig. S1). However, none of the curves reached a plateau, indicating that the observed level of richness (unique operational taxonomic unit), as determined by the unique sequences and overall sampling intensity was insufficient to fully describe the richness of rumen bacterial communities.

Sequences were then grouped according to diet in order to determine the effect of forage, oilseed and the forage by oilseed interaction and to account for low sequence abundance in some individual samples. A distance matrix was constructed using the average neighbour algorithm at 0.05 (genus) and 0.25 (phylum) phylogenetic distances to determine the most accurate phylogenetic tree structure. Pairwise distances between aligned sequences were calculated at a 0.97% similarity cut off and then clustered into unique OTUs (operational taxonomic unit). Any sequences aligning for more than 97% of the sequence were considered to be from the same bacterial species (OTU). In total, there were 64,396 quality reads with an average of 16,099±6731 reads and an average of 364 unique OTUs per diet. Mothur was also used to calculate the coverage for each treatment (Fig. S1), and to create a dendrogram based on treatment differences using OTU dissimilarity between the structures of two communities [26]. Calculations of percentage of sequences within taxonomic classifications at the genus level were performed using a custom summation script [27].

### Subcutaneous fatty acid analysis

Subcutaneous fat samples (50 mg) were freeze-dried and directly methylated with sodium methoxide [28]. As an internal standard, 1 ml of 1 mg *c*10-17:1 methyl ester/ml toluene (standard no. U-42M form Nu-Check Prep Inc., Elysian, MN, USA) was added prior to addition of methylating reagents. Fatty acid methyl esters (FAME) were analysed by gas chromatography using a CP-Sil88 column (100 m, 25 µm ID, 0.2 µm film thickness) in a CP-3800 gas chromatograph equipped with an 8600-series autosampler (Varian Inc., Walnut Creek, CA, USA). Two gas chromatography (GC) analyses were conducted per sample using complementary temperature programs with 150°C and 175°C plateaus according to Kramer, et al. [29]. CLA isomers not separated by GC were further analysed using Ag^+^-HPLC as described by Cruz-Hernandez et al. [30].

For the identification of FAME by GC, the reference standard no. 601 from Nu-Check Prep Inc, Elysian, MN, USA was used. Branched-chain FAME were identified using a GC reference standard BC-Mix1 purchased previously from Applied Science (State College, PA, USA). For CLA isomers, the UC-59M standard from Nu-Chek Prep Inc. was used which contains all four positional CLA isomers. *Trans*-18:1, CLA isomers and other BH products not included in the standard mixtures were identified by their retention times and elution orders as reported in literature [29]–[31]. The FAME were quantified, using chromatographic peak area and internal standard based calculations. Only FAME representing more than 0.01% of total FAME were included in tables and figures with the exception of BH products, where all the quantified isomers were reported.

### Statistical analysis

All data were analysed, using the PROC MIXED procedure of SAS [32]. The statistical model for FA profiles and genus percent abundance included the fixed effects of oilseed, forage and oilseed × forage interaction,with slaughter date and pen considered as random effects and animal as the experimental unit. Since the random effect of pen nested within the oilseed × forage interaction was not significant, it was removed from the model. Treatment means were generated and separated, using the LSMEANS and PDIFF options respectively [32].

For the comparison of high and low levels of VA within diet to bacterial abundance using 2×2×2 factorial analysis, there was a significant FA level by oilseed type interaction. Therefore, data were reanalyzed as 2×4 factorial ANOVA comparing forage to high and low levels of a FA for each oilseed. For the analysis, treatment means were generated and separated, using Tukey-Kramer and PDIFF options [32]. To relate rumen bacterial profiles to FA profiles, percent abundance of genus level taxa were additionally analyzed in pairwise Pearson correlation to FA data, using the PROC CORR procedure of SAS [32]. The significance threshold for all statistical analyses was set at *P*<0.05.

## Results

### Animal performance

Data on animal performance are detailed in a companion paper by Mapiye et al [19]. In summary, steers fed SS diets (13.2±0.37) had higher (*P*<0.05) DM intake than those fed FS (12.1±0.37), and steers fed GH consumed more (13.3±0.37; *P*<0.05) feed than those fed RC (12.1±0.37). As a result, average daily gain and final live weights were higher (*P*<0.05) in steers fed SS (0.7 kg/d±0.08 and 550 kg±9.55) compared to FS (0.51 kg/d±0.08 and 503 kg±9.55) and final live weights were also higher (*P*<0.05) in steers fed GH (551 kg±9.55), as compared to those fed RC (517 kg±9.55). Steers fed SS diets (7.66±0.38 mm) had a tendency to have thicker (P = 0.08) final subcutaneous fat depth than steers fed FS diets (6.69±0.38 mm), but forage type and its interaction with oilseed had no effect (*P*>0.05) on final subcutaneous fat thickness.

### Subcutaneous fatty acid profiles

The proportions of total PUFA in subcutaneous fat were affected by oilseed type with steers fed SS diets having greater (*P*<0.05) proportions than those fed FS (Table 2). Oilseed type also influenced the proportions of total and individual *n*-6 PUFA (18:2*n*-6, 20:3*n*-6, 20:4*n*-6), with steers fed SS having greater (*P*<0.05) proportions in subcutaneous fat than steers fed FS. For all diets, LA was the most prominent *n*-6 PUFA, accounting for more than 90% of total *n*-6 PUFA.

**Table 2:** Effect of forage type and oilseed interaction on fatty acid profiles of subcutaneous fat from beef steers.

	Grass hay		Red clover			P-value		
Variable	Flax	Sunflower	Flax	Sunflower	SEM	Oilseed	Forage	O*F^1^
∑ PUFA	1.97	2.10	1.80	2.11	0.07	0.001	0.25	0.19
∑ *n*-6	1.40	1.75	1.29	1.82	0.06	<0.001	0.78	0.15
18:2*n*-6	1.30	1.63	1.20	1.70	0.06	<0.001	0.81	0.17
20:2*n*-6	0.03	0.03	0.03	0.03	0.00	0.10	0.18	0.50
20:3*n*-6	0.04	0.05	0.03	0.05	0.00	<0.001	0.26	0.27
20:4*n*-6	0.03	0.04	0.03	0.05	0.00	<0.001	0.65	0.18
∑ *n*-3	0.58	0.35	0.51	0.29	0.02	<0.001	0.001	0.90
18:3*n*-3	0.50	0.30	0.44	0.24	0.02	<0.001	0.001	0.88
20:3*n*-3	0.02	0.01	0.02	0.01	0.00	<0.001	0.25	0.72
22:5*n*-3	0.06	0.04	0.05	0.04	0.00	<0.001	0.03	0.81
∑ CLNA	0.29^a^	0.08^b^	0.32^a^	0.05^b^	0.01	<0.001	0.90	0.03
*c*9,*t*11,*t*15-18:3	0.10	0.03	0.11	0.02	0.01	<0.001	0.66	0.16
*c*9,*t*11,*c*15-18:3	0.19^a^	0.05^b^	0.21^a^	0.03^b^	0.01	<0.001	0.71	0.04
∑ AD	2.61	1.68	2.35	1.17	0.09	<0.001	<0.001	0.17
∑ CLA	2.10	2.47	2.07	2.34	0.18	0.02	0.55	0.72
*∑ c,t*-CLA	1.91	2.37	1.90	2.26	0.18	0.001	0.65	0.73
∑ *t,t*-CLA	0.19	0.10	0.17	0.09	0.01	<0.001	0.06	0.95
∑ *t*-18:1	6.14	8.20	5.51	7.66	0.38	<0.001	0.09	0.90
∑ *c*-MUFA	45.2	44.7	46.0	43.5	1.09	0.16	0.85	0.35
*c*9-14:1	1.47	1.55	1.75	1.34	0.14	0.25	0.79	0.09
*c*7-16:1	0.20	0.19	0.22	0.22	0.01	0.42	0.001	0.74
*c*9-16:1	5.12	4.77	5.95	4.68	0.32	0.02	0.26	0.16
*c*9-17:1	0.78	0.68	0.83	0.71	0.03	0.001	0.27	0.76
*c*9-18:1	34.3	34.0	34.3	33.7	0.73	0.45	0.80	0.82
*c*11-18:1	1.33	1.11	1.32	1.12	0.09	0.02	0.97	0.88
*c*12-18:1	0.62^c^	1.38^a^	0.36^d^	0.81^b^	0.05	<0.001	<0.001	0.001
*c*13-18:1	0.44	0.38	0.47	0.34	0.03	0.01	0.90	0.29
*c*14-18:1	0.09	0.09	0.08	0.07	0.00	0.54	<0.001	0.43
*c*15-18:1	0.49	0.23	0.39	0.18	0.02	<0.001	<0.001	0.16
*c*16-18:1	0.08	0.08	0.07	0.07	0.00	0.10	0.06	0.70
*c*10-19:1	0.08	0.04	0.08	0.04	0.00	<0.001	0.19	0.53
*c*9-20:1	0.12	0.11	0.12	0.11	0.00	0.01	0.70	0.47
*c*11-20:1	0.15	0.13	0.15	0.14	0.01	0.23	0.67	0.40
∑ BCFA	2.01	1.83	1.93	1.90	0.04	0.01	0.86	0.07
*iso*-14:0	0.07	0.06	0.07	0.07	0.001	0.75	0.45	0.38
*iso*-15:0	0.24	0.22	0.22	0.22	0.01	0.07	0.16	0.07
*anteiso*-15:0	0.28	0.27	0.28	0.29	0.01	0.83	0.79	0.28
*iso*-16:0	0.29	0.27	0.29	0.30	0.01	0.80	0.16	0.24
*iso*-17:0	0.34	0.31	0.32	0.31	0.01	0.01	0.05	0.12
*anteiso*-17:0	0.64	0.57	0.61	0.59	0.01	0.001	0.88	0.07
*iso*-18:0	0.15	0.12	0.15	0.13	0.00	<0.001	0.89	0.32
∑ SFA	38.6	37.9	38.9	40.3	1.07	0.75	0.19	0.28
14:0	3.45	3.24	3.51	3.36	0.14	0.17	0.47	0.83
15:0	0.59	0.55	0.63	0.61	0.02	0.06	0.001	0.61
16:0	22.8	22.1	23.8	23.1	0.50	0.06	0.02	0.97
17:0	0.75^a^	0.68^c^	0.70^b^	0.75^a^	0.03	0.72	0.60	0.03
18:0	10.8	11.1	10.1	12.4	0.71	0.08	0.74	0.17
19:0	0.05	0.05	0.04	0.05	0.00	0.68	0.42	0.39
20:0	0.08	0.09	0.08	0.10	0.01	0.12	0.28	0.38
22:0	0.02	0.02	0.02	0.02	0.00	0.10	0.54	0.08

a,b,c,d Means with different superscripts for a particular fatty acid profile have a significant oilseed × forage interaction(P<0.05); SEM, standard error of mean;

1 Oilseed type × forage type interaction; *c*, *cis*; *t*, *trans*;

**∑** PUFA, sum of polyunsaturated fatty acids = ∑ *n*-6 +∑ *n*-3; ∑ *n*-6 = sum of 18:2*n*-6, 20:2*n*-6, 20:3*n*-6, 20:4*n*-6; ∑ *n*-3 sum of 18:3*n*-3, 20:3*n*-3, 22:5*n*-3; ∑CLNA, sum of conjugated α-linolenic acid = *c*9,*t*11,*t*15-, *c*9,*t*11,*c*15-; **∑**AD, total atypical dienes = sum of *t*11,*t*15-, *c*9,*t*13-/*t*8,*c*12-, *t*8,*c*13-, *c*9,*t*12-/*c*16-18:1, *t*9,*c*12-, *t*11,*c*15-, *c*9,*c*15-, *c*12,*c*15-; ∑ CLA, conjugated linoleic acid = sum of *t,t*-CLA + sum of *c,t*-CLA; ∑ *trans-trans*-CLA = sum of *t*12,*t*14-, *t*11,*t*13-, *t*10,*t*12-, *t*9,*t*11-, *t*8,*t*10-, *t*7,*t*9- *t*6,*t*8-; ∑ *cis-/trans*-CLA = sum of *c*9,*t*11-, *t*7,*c*9-, *t*11,*c*13-, *t*12,*c*14-, *c*11,*t*13-, *t*10,*c*12-, *t*8,*c*10-, *t*9,*c*11-; ∑ *t*-18:1, sum of *trans*-18:1 isomers = *t*6,*t*7,*t*8-, *t*9-, *t*10-, *t*11-, *t*12-, *t*13,*t*14-, *t*15-, *t*16-; ∑ *c*-MUFA = sum of *c*9-14:1, *c*7-16:1, *c*9-16:1, *c*11-16:1, *c*9-17:1, *c*9-18:1, *c*11-18:1, *c*12-18:1, *c*13-18:1, *c*14-18:1, *c*15-18:1, *c*9-20:1, *c*11-20:1; ∑ BCFA, branched chain fatty acids = sum of *iso*-15:0, *anteiso*15:0, *so*16, *iso*17:0, *anteiso*17:0, *iso*18:0; ∑ SFA = sum of 14:0, 15:0, 16:0, 17:0, 18:0, 19:0, 20:0, 22:0.

For *n*-3 PUFA, diets containing FS as opposed to SS, and GH as opposed to RC exhibited elevated (*P*<0.05) proportions of total *n*-3 PUFA, 18:3*n*-3 (ALA) and 22:5*n*-3 (Table 2), but in general, increases were influenced more by oilseed than forage type. Alpha-linolenic acid was the most abundant *n*-3 PUFA, making up over 80% of total *n*-3 PUFA in all diets.

An oilseed × forage type interaction was detected for total conjugated linolenic acids (CLNA) and *c*9,*t*11,*c*15-18:3, but upon means separation only an effect of oilseed was detected with FS diets resulting in greater (*P*<0.05) proportions than SS diets (Table 2). The proportions of *c*9,*t*11,*t*15-18:3 were also only influenced by oilseed type, with steers fed FS having greater (*P*<0.05) proportions than those fed SS.

**Figure 1:**
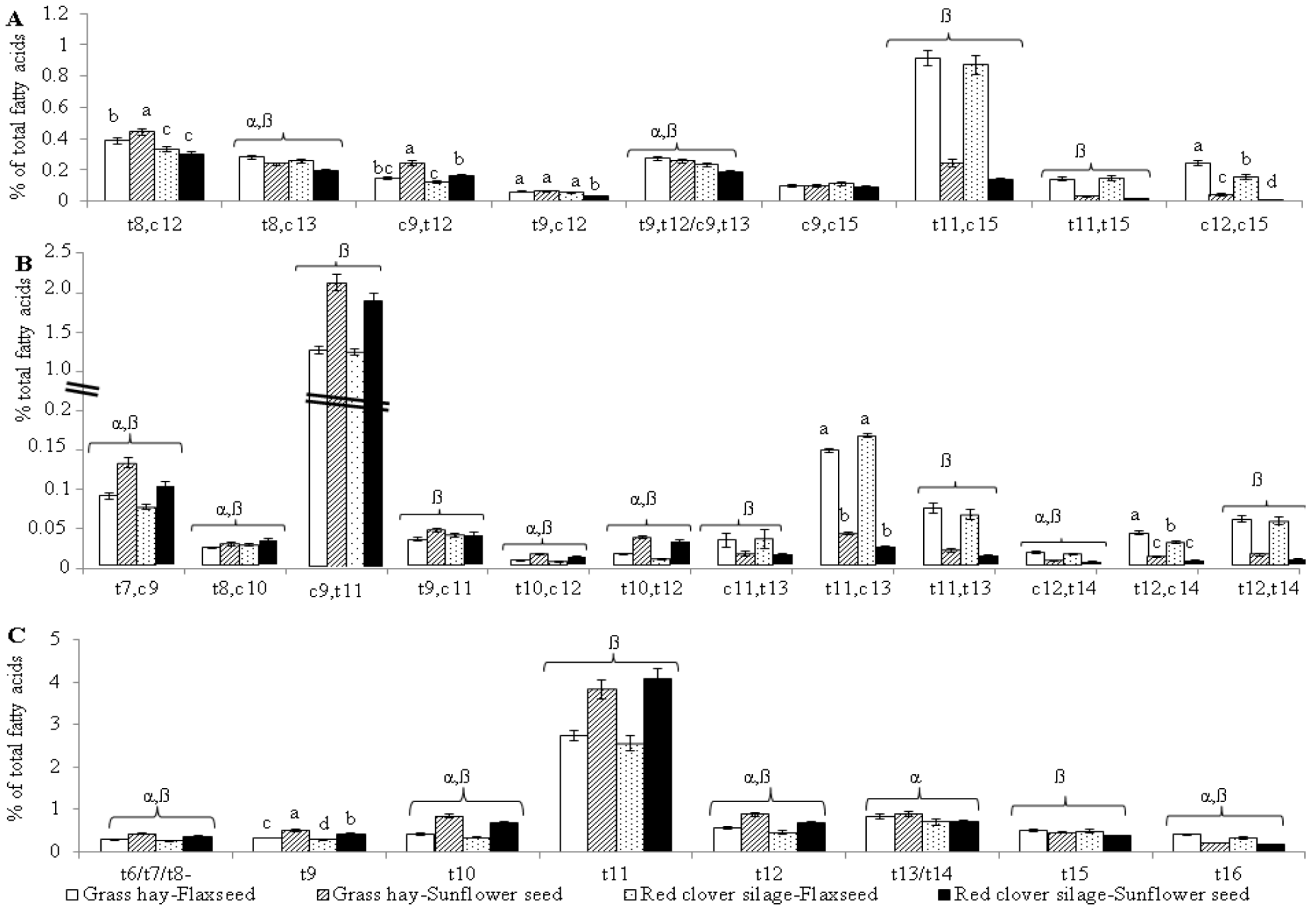
Effect of forage type and oilseed supplementation on atypical dienes (A), conjugated linoleic acid (B) and *trans*-18:1 isomers (C) in subcutaneous fat of beef steers. ^a,b,c,d^ Means (± standard error) with different superscripts for a particular fatty acid profiles are significantly different (*P*<0.05). α: Significant forage effect (*P*<0.05); β: Significant oilseed effect (*P*<0.05).

Based on the type of oilseed and forage fed, two AD isomer patterns were observed (Fig. 1A). The proportions of AD isomers likely largely derived from ALA (*t*8,*c*13-; *t*9,*t*12-/*c*9,*t*13-; *t*11,*c*15-; *t*11,*t*15-; *c*12,*c*15-18:2) were increased by FS (P<0.05). Of the isomers in this group, minor forage effects were found for *t*8,*c*13- and *t*9,*t*12-/*c*9,*t*13-18:2 (*P*<0.05), with diets containing GH resulting in slightly increased (*P*<0.05) proportions than diets containing RC. A small but significant forage × oilseed type interaction (P<0.05) was found for *c*12,*c*15-18:2 with the GH-FS diet resulting in the greatest proportions followed by RC-FS, GH-SS and RC-SS diets, respectively. For AD likely largely derived from LA (*t*8, *c*12-; *c*9,*t*12-; *t*9,*c*12-18:2), forage × oilseed type interactions were found (*P*<0.05) with amounts being greatest for the GH-SS diet. The dominant AD isomer irrespective of diet was *t*11, *c*15-18:2 accounting for up to 25% and 35% of total AD in steers fed SS and FS, respectively.

For CLA, two clear isomer patterns were found based on the type of oilseed fed (Fig. 1B). The proportions of CLA isomers with the first double bond at carbon 10 or closer to the carboxyl end (*t*7,*c*9-; *t*8,*c*10-; *c*9,*t*11- (RA); *t*9,*c*11-; *t*10,*c*12-; *t*10,*t*12-18:2) were more elevated (*P*<0.05) when feeding SS as compared to FS. Some minor forage effects were also found for *t*7,*c*9-; *t*8,*c*10-; *t*10,*c*12- and *t*10,*t*12-18:2 with diets containing GH v*s.* RC yielding slightly greater proportions. The proportions of CLA isomers with the first double bond at carbon 11 or further from the carboxyl end (*c*11,*t*13-; *t*11,*c*13-; *t*11,*t*13-; *c*12,*t*14-; *t*12,*t*14-; *t*12,*c*14-18:2) were mostly increased (*P*<0.05) by inclusion of FS. Minor forage effects were also noted for *c*12, *t*14-18:2 (*P*<0.05) while forage × oilseed type interactions were noted for *t*11,*c*13- and *t*12, *c*14-18:2 (*P*<0.05). For all diets, RA accounted for over 70% of total CLA (Fig. 1B).

For *t*-18:1, an isomer pattern was found based on the type of oilseed and forage fed (Fig. 1C). The proportions of *t*-18:1 isomers with double bonds from carbon 6 to 12 were primarily greater (*P*<0.05) with SS than FS, and for the majority of these isomers, feeding GH *vs.* RC diets also led to increases (*P*<0.05), but at a reduced magnitude as compared to oilseeds. For *t*-18:1 isomers with double bonds from carbon 13 to 16, the pattern of differences was less strongly linked to forage or oilseed effects (Fig. 1C). Feeding diets containing FS as opposed to SS increased (*P*<0.05) the proportions *t*15- and *t*16-18:1 (Fig. 1C). The proportions of *t*13-/*t*14- and *t*16-18:1 were elevated (*P*<0.05) by diets containing GH as opposed to RC. Vaccenic acid was the predominant *t*-18:1 isomer and accounted for 45% and 50% of total *t*-18:1 isomers in the subcutaneous fat of steers fed FS and SS diets, respectively (Fig. 1C).

Overall, feeding diets containing FS as opposed to SS elevated (*P*<0. 05) the proportions of several individual *c*-monounsaturated fatty acids (MUFA) isomers (*c*9-16:1, *c*9-17:1, *c*11-18:1, *c*13-18:1, *c*15-18:1, *c*10-19:1, *c*9-20:1). An oilseed type × forage type interaction influenced the proportions of *c*12-18:1, with steers fed GH-SS having the largest proportions followed by those fed RC-SS, GH-FS and RC-FS, respectively (*P*<0.05; Table 2). Relative to feeding RC, GH reduced (*P*<0.05) the proportions of *c*7-16:1 and increased (*P*<0.05) the proportions of *c*14- and *c*15-18:1 (Table 2). Oleic acid (*c*9-18:1) was the dominant MUFA isomer, accounting for over 75% of total *c*-MUFA in all diets (Table 2).

Feeding diets containing FS compared to SS increased total branched-chain fatty acids (BCFA; *P*<0.05) as a result of increases in the proportions of *iso*-17:0, *anteiso*-17:0 and *iso*-18:0 (*P*<0.05) (Table 2). Neither oilseed nor forage type had any effect on total SFA (*P*<0.05), but proportions of 15:0, 16:0 and *iso*-17:0 were influenced by forage type, with steers fed RC having higher (*P*<0.05), 15:0, 16:0 and lower (*P*<0.05) *iso*-17:0 proportions than those fed GH. An oilseed type × forage type interaction was observed for steers fed GH-FS and RC-SS,,with steers fed RC-FS having intermediate and steers fed GH-SS having the lowest proportions of 17:0 (*P*<0.05). Palmitic acid (16:0) was the most abundant saturated FA (SFA), constituting about 60% of the total SFA in subcutaneous fat of steers across all diets.

### Rumen bacterial profiles

**Figure 2:**
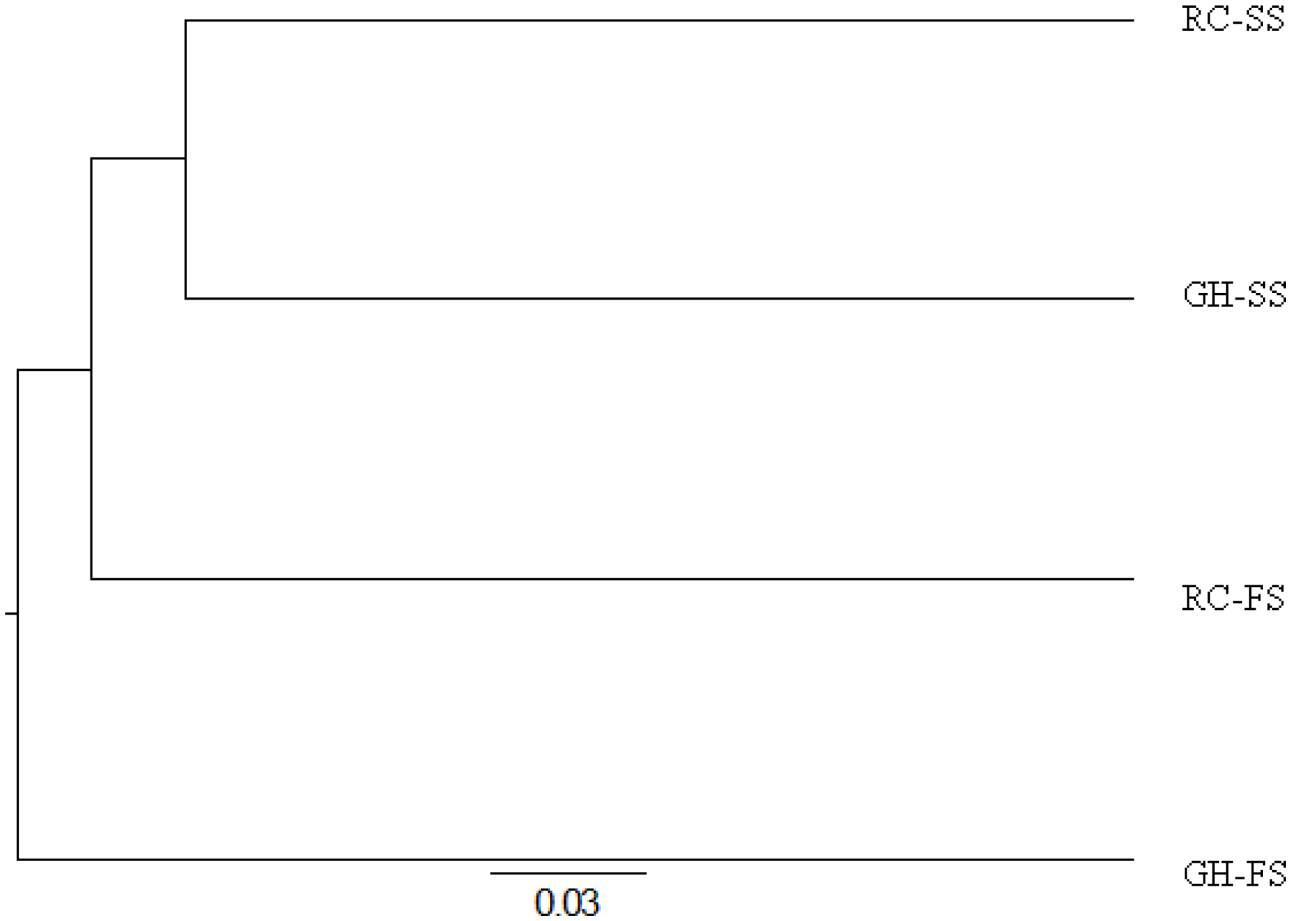
Dendrogram showing distances between dietary treatments based on similarity of sequences using Jaccard analysis (GH-SS: hay-sunflower seed, GH-FS: hay-flax seed, RC-SS: red clover-sunflower seed, RC-FS: red clover-flax seed).

**Figure 3:**
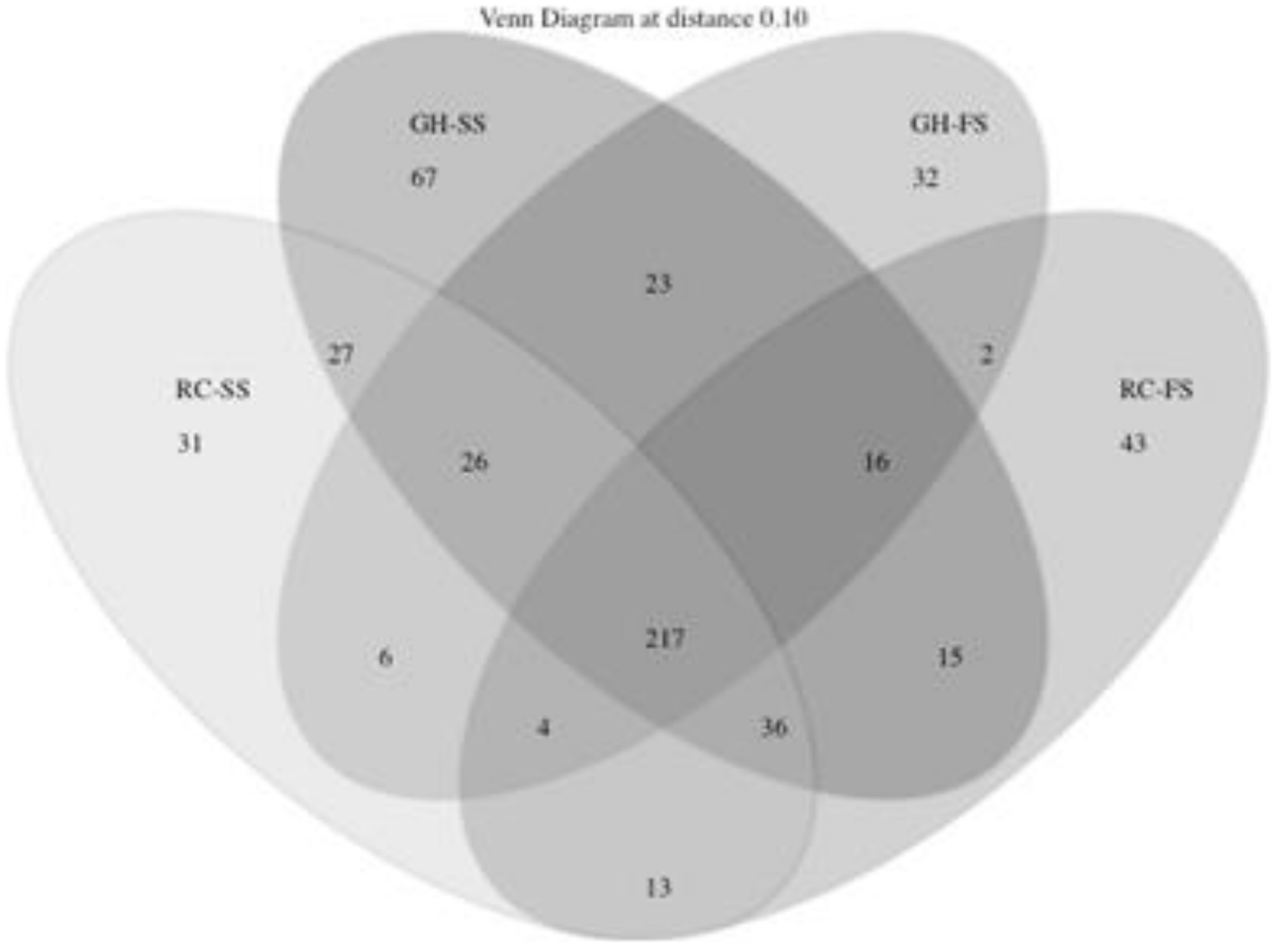
Venn diagram showing OTU's unique to and shared by each of the dietary treatments (GH-SS: hay-sunflower seed, GH-FS: hay-flax seed, RC-SS: red clover-sunflower seed, RC-FS: red clover-flax seed).

The Yue and Clayton [26] measure of dissimilarity among communities was used to create a dendrogram showing the separation of OTU in samples from individual diets (Fig. 2). Despite clustering of diets based on type of forage, diets were not found to differ (*P* = 1.0). The OTU's calculated for each diet were used to construct a Venn diagram (Fig. 3), which identified a total of 558 OTU's across the 4 diets. The number of OTU's that were associated with each diet ranged from 326 to 427. Of these OTU's, 217 were shared by all 4 diets. Using non-parametric estimators in Mothur, it was predicted that the core microbiome was composed of 256 OTU's.

Using a summation script, the percent abundance of each of the 87 classified genera were determined and those which differed (P<0.05) by forage or oilseed type are listed in Table 3. Of these genera, 12 were impacted by forage type, 9 were impacted by oilseed type and 5 exhibited a forage × oilseed interaction. The taxa *Butyrivibrio*, and *Syntrophococcus* were higher (*P*<0.05) in the GH diets, whereas *Fibrobacter was* higher (*P*<0.01) in RC diets. *Blautia*, *Eubacterium*, and *Olsenella* were higher in the GH-FS diet and *Anaerophaga* was lowest in GH fed cattle. *Johnsonella* was highest with GH-SS, whereas *Mogibacterium* and *Wandonia* were highest in cattle fed RC-FS. When comparing oilseed supplementation, *Acidaminobacter* was the only taxon that was consistently higher in cattle fed FS. *Barnesiella* was abundant across all diets, but it was higher (*P*<0.05) in the GH-FS diet and lowest with GH-SS. *Marvinbryantia* exhibited the opposite response being more abundant (*P*<0.02) with GH-SS than with GH-FS. *Bellilinea*, *Blautia*, *Guggenheimella*, *Nubsella* and *Sporobacter* were all influenced by an oilseed × forage interaction. Of these, *Bellilnea*, *Guggenheimella*, and *Nubsella* were present in greater abundance (*P*<0.05) in the RC-FS fed cattle.

**Table 3:** Percent abundance of genera impacted by forage type (F), oilseed type included in the diet (O), level of vaccenic acid (VA) in backfat or any interaction of these factors.

	Hay				Red Clover											
	Flax		Sunflower		Flax		Sunflower			*P*-value for indicated factor						
Bacterial Genera	High VA^1^	Low VA	High VA	Low VA	High VA	Low VA	High VA	Low VA	SEM	F	O	VA	F × O	F × VA	O × VA	F × O × VA
*Acidaminobacter*	0.00	0.37	0.00	0.00	0.17	0.17	0.00	0.00	0.11	0.93	0.03	0.24	0.93	0.24	0.24	0.24
*Anaerophaga*	0.76	1.86	0.92	1.82	1.99	2.21	1.90	2.66	0.40	0.01	0.68	0.02	0.85	0.38	0.77	0.52
*Anaerosporobacter*	0.61	0.42	0.61	0.71	0.20	0.54	0.37	0.00	0.19	0.04	0.91	0.83	0.24	0.90	0.47	0.09
*Anaerostipes*	0.18	0.22	0.18	0.00	0.47	0.73	0.00	0.23	0.17	0.10	0.03	0.49	0.15	0.21	0.63	0.70
*Asaccharobacter*	0.46	0.00	0.17	0.00	0.00	0.00	0.00	0.00	0.10	0.05	0.35	0.05	0.35	0.05	0.35	0.35
*Asteroleplasma*	0.00^b^	0.00^b^	0.83^a^	0.21^a^ ^b^	0.20^a^ ^b^	0.00^b^	0.00^b^	0.43^a^ ^b^	0.15	0.34	0.01	0.37	0.07	0.06	0.98	0.01
*Blautia*	1.48	1.31	1.19	0.99	0.47	0.66	1.34	0.89	0.23	0.02	0.45	0.35	0.02	0.87	0.31	0.36
*Butyrivibrio*	2.75	2.64	2.56	2.20	1.26	1.54	1.68	1.46	0.29	0.00	0.73	0.62	0.25	0.54	0.38	0.77
*Clostridium_IV*	0.88	0.70	1.09	1.13	0.77	0.83	1.47	1.50	0.25	0.30	0.01	0.96	0.33	0.76	0.79	0.73
*Eubacterium*	1.02	0.76	0.56	0.61	0.23	0.40	0.28	0.23	0.21	0.01	0.25	0.88	0.42	0.59	0.89	0.39
*Faecalibacterium*	0.00^b^	0.26^a^ ^b^	0.47^a^ ^b^	0.00^b^	0.68^a^	0.23^a^ ^b^	0.35^a^ ^b^	0.60^a^	0.23	0.10	0.70	0.55	0.82	0.99	0.96	0.04
*Fibrobacter*	0.59	1.89	0.37	0.98	2.06	2.18	1.48	2.85	0.38	0.00	0.36	0.01	0.28	0.70	0.62	0.09
*Galbibacter*	0.4^c^	2.55^c^	2.6^c^	2.8^b^ ^c^	3.41^a^	3.71^b^ ^c^	1.99^c^	3.74^a^ ^b^	0.11	0.00	0.52	0.44	0.10	0.77	0.00	0.00
*Guggenheimella*	0.40	2.55	2.60	2.63	2.61	3.49	1.99	3.09	0.50	0.05	0.38	0.01	0.03	0.89	0.20	0.12
*Hallella*	2.18^a^	1.47^a^ ^b^	1.19^a^ ^b^	1.22^a^ ^b^	1.54^a^ ^b^	2.25^a^	1.39^a^ ^b^	1.08^b^	0.30	0.82	0.01	0.73	0.93	0.22	0.73	0.05
*Johnsonella*	0.83	0.37	0.98	0.72	0.37	0.00	0.33	0.23	0.30	0.02	0.37	0.13	0.69	0.74	0.53	0.92
*Marvinbryantia*	1.43	0.55	1.88	1.30	1.38	1.12	1.66	1.21	0.24	0.78	0.03	0.01	0.24	0.28	0.88	0.49
*Mogibacterium*	0.17	0.18	0.40	0.00	0.88	0.71	0.70	0.50	0.17	0.00	0.48	0.13	0.35	0.95	0.37	0.43
*Nubsella*	0.27^b^	1.45^a^ ^b^	1.25^a^ ^b^	1.13^a^ ^b^	2.16^a^	1.45^a^ ^b^	0.85^a^ ^b^	1.50^a^ ^b^	0.28	0.03	0.44	0.22	0.03	0.17	0.93	0.00
*Olsenella*	0.48	0.20	0.18	0.00	0.00	0.00	0.00	0.00	0.10	0.03	0.19	0.22	0.19	0.22	0.77	0.77
*Oscillibacter*	0.00	0.61	0.60	0.76	0.54	0.53	0.80	0.79	0.19	0.21	0.03	0.18	0.68	0.16	0.43	0.42
*Paludibacter*	0.00	0.79	0.24	0.75	1.04	0.79	0.56	1.12	0.20	0.01	0.94	0.01	0.55	0.10	0.36	0.08
*Phocaeicola*	2.33^a^	1.23^a^ ^b^	1.06^a^ ^b^	1.57^a^ ^b^	1.05^a^ ^b^	1.88^a^ ^b^	1.39^a^ ^b^	0.62^b^	0.31	0.17	0.05	0.55	0.99	0.46	0.99	0.00
*Pseudo-sphingobacterium*	1.69^b^	5.94^a^	1.99^b^	4.38^a^ ^b^	6.7^a^	5.64^a^	4.52^a^ ^b^	6.14^a^	0.70	0.00	0.16	0.00	0.84	0.01	0.68	0.04
*Pseudozobellia*	0.18	0.54	0.00	0.00	0.00	0.41	0.00	0.19	0.15	0.80	0.05	0.04	0.26	0.57	0.21	0.76
*Sporobacter*	1.21	0.76	1.12	1.49	1.16	1.30	0.97	0.90	0.86	0.42	0.66	0.57	0.05	0.51	0.49	0.12
*Syntrophococcus*	2.31	1.50	2.28	1.55	1.36	1.35	1.61	1.08	0.23	0.00	0.99	0.01	0.94	0.14	0.51	0.37
*Treponema*	1.95^b^	2.62^a^ ^b^	2.56^a^ ^b^	2.11^a^ ^b^	2.52^a^ ^b^	2.14^a^ ^b^	2.06^a^ ^b^	2.77^a^	0.23	0.82	0.79	0.60	0.95	0.92	0.98	0.05
*Wandonia*	0.00	0.29	0.19	0.00	0.32	0.70	0.34	0.51	0.23	0.05	0.70	0.33	0.92	0.49	0.30	0.67

a,b,c Means with different superscripts within a row are significantly different (P<0.05) based on the three-way interaction.

1 VA, Vaccenic acid; F, Forage; O, Oilseed; SEM, standard error of the mean.

Additionally, Pearson correlation was used to relate the percent abundance of measured bacterial taxa to measured subcutaneous FA. The highest correlation (*P*<0.004) between percent abundance and FA profile was for the genus *Clostridium IV* (Table 4). *Clostridium IV* was highly correlated to total *n*-3 PUFA (*P*<0.004; R^2^ = 0.32), total CLNA (*P*<0.001; R^2^ = 0.38) and total atypical dienes (*P*<0.001; R^2^ = 0.40). The only other genera, which was highly correlated with specific FA profiles, was *Fibrobacter*, which correlated to total CLA (*P*<0.001; R^2^ = 0.40) and *Marvinbryantia*, which correlated to total *t*MUFA (*P*<0.002; R^2^ = 0.36). Additionally, two bacterial genera, *Acidaminobacter* and *Asteroleplasma* were highly (*P*<0.001) correlated to specific individual FA. *Acidaminobacter* was correlated to FA *iso*-18:0 with a R^2^ = 0.39 and *Asteroleplasma* was correlated to *c*12-18:1 with a R^2^ = 0.44.

**Table 4:** Correlation of bacterial genera to subcutaneous fatty acid groupings.

		Fatty Acid Group						
Genera Taxa		Total n-6 and n-3	Total n-6	Total n-3	Total CLNA	Total AD	Total CLA	Total trans MUFA
*Acidaminobacter*	P-value	0.75	0.31	0.05	0.07	0.26	0.33	0.03
	R^2^	0.00	0.05	0.17	0.14	0.06	0.04	0.19
	Corr.^1^			+				−
*Anaerostipes*	P-value	0.03	0.02	0.57	0.16	0.56	0.36	0.03
	R^2^	0.20	0.22	0.01	0.09	0.02	0.04	0.19
	Corr.	−	−					−
*Asteroleplasma*	P-value	0.20	0.11	0.32	0.17	0.53	0.14	0.05
	R^2^	0.07	0.11	0.04	0.08	0.02	0.09	0.17
	Corr.							+
*Clostridium_IV*	P-value	0.39	0.06	0.004	0.001	0.001	1.00	0.38
	R^2^	0.03	0.15	0.32	0.38	0.40	0.00	0.04
	Corr.			−	−	−		
*Ethanoligenens*	P-value	0.97	0.43	0.03	0.04	0.09	0.48	0.33
	R^2^	0.00	0.03	0.19	0.19	0.12	0.02	0.04
	Corr.			−	−			
*Fibrobacter*	P-value	0.37	0.54	0.50	0.87	0.33	0.001	0.06
	R^2^	0.04	0.02	0.02	0.00	0.04	0.40	0.15
	Corr.						−	
*Galbibacter*	P-value	0.20	0.25	0.83	0.52	0.66	0.01	0.31
	R^2^	0.07	0.06	0.00	0.02	0.01	0.25	0.05
	Corr.						−	
*Marvinbryantia*	P-value	0.30	0.18	0.39	0.30	0.45	0.08	0.002
	R^2^	0.05	0.08	0.03	0.05	0.03	0.13	0.36
	Corr.							+
*Olsenella*	P-value	0.54	0.28	0.17	0.26	0.04	0.26	0.49
	R^2^	0.02	0.05	0.08	0.06	0.18	0.06	0.02
	Corr.					+		
*Pseudo-butyrivibrio*	P-value	0.42	0.43	0.97	0.74	0.64	0.03	0.38
	R^2^	0.03	0.03	0.00	0.01	0.01	0.20	0.03
	Corr.						+	

1 Positive (+) or negative (−) correlation (Corr.) for P<0.05.

This difference in VA levels coincided with changes in the percent abundance of several genera including *Anaerophaga*, *Asaccharobacter*, *Fibrobacter*, *Guggenheimella*, *Marvinbryantia*, *Paludibacter*, *Pseudosphingobacterium*, *Pseudozobellia* and *Syntrophococcus* (Table 5).

**Table 5:** Changes in composition of bacterial abundance in animals with high *vs.*low vaccenic acid in the backfat.

	Vaccenic Acid within Treatment			
	High (n = 12)	Low (n = 12)-	SEM	P-value
Vaccenic Acid (% of total FAME)^1^	4.44	2.44	0.09	
				
Bacterial Abundance (% of total)				
*Anaerophaga*	1.39^b^	2.14^a^	0.02	0.02
*Asaccharobacter* 2	0.02	0.00	0.01	0.05
*Fibrobacter*	1.13^b^	1.97^a^	0.02	0.01
*Guggenheimella*	1.90^b^	2.94^a^	0.02	0.01
*Marvinbryantia*	1.59^a^	1.04^b^	0.01	0.01
*Paludibacter*	0.46^b^	0.87^a^	0.01	0.01
*Pseudosphingobacterium* 2 ^,^ 3	3.73	5.53	0.04	<0.01
*Pseudozobellia*	0.05^b^	0.28^a^	0.01	0.04
*Syntrophococcus*	1.89^a^	1.37^b^	0.01	0.01

1 Significant two-way interaction of vaccenic acid level × oilseed (P<0.05).

2 Significant two-way interaction of vaccenic acid level × forage (P<0.05).

3 Significant three-way interaction of vaccenic acid level × oilseed × forage (P<0.05).

a,b Means with different superscripts within a row are significantly different (P<0.05).

## Discussion

### Animal performance

The finding that steers fed diets containing FS grew slightly slower and had lower final live weight than steers fed diets containing SS may be related to palatability issues resulting from the addition of FS [33] and/or the inclusion of ground straw [34] in FS diets to balance for digestible energy across oilseed diets. The explanations for the lower final live weight observed, when feeding RC as opposed to GH are less clear, but the quality of silage fed and higher crude fat content of RC diets may have been partially responsible. Silage quality (dry matter content and fermentation quality) was not assessed in this experiment, but fat levels greater than 5% have been reported to inhibit ruminal fiber digestion, increasing rumen fill and reducing DM intake [35].

### Subcutaneous fatty acid profiles

The elevated levels of total PUFA in the subcutaneous fat of steers fed diets containing SS was related to the increase in *n*-6 PUFA, especially LA. This resulted from the large proportion of LA in diets containing SS. In most diets, the extent of LA BH is lower than that of ALA [36] because greater levels of LA in the rumen inhibits the growth of many of the bacterial species involved in BH [37]. This inhibitory effect results in increased tissue deposition of LA and its long chain *n*-6 PUFA derivatives when compared to ALA and its long chain *n*-3 PUFA derivatives [38]. Additionally, the physical form of the oilseed fed (whole SS *vs.* triple rolled FS) might have also influenced BH in the rumen. Rolling has been reported to enhance the availability of PUFA in the rumen, thereby increasing the degree to which they are BH [39]. The increase in surface area as a result of rolling would have increased microbial attachment and therefore potentially increased the rate of lipolysis and BH. The effect of forage on FA profiles was confounded by the physical form of the feed. The lack of accumulation of PUFA in cattle fed RC diets was unexpected as it is well known that RC has increased levels of poly-phenol oxidase (PPO) which increases PUFA levels in meat and milk [11]. It is possible that the activity of PPO was reduced during ensiling.

As anticipated, feeding diets containing FS, a rich source of ALA, increased the deposition of ALA and its long chain derivatives, 20:3*n*-3 and 20:5*n*-3 in subcutaneous fat. This is likely due to part of dietary ALA escaping ruminal BH and therefore being available for direct deposition in the tissue. The reason for the increased *n*-3 PUFA proportions observed in steers fed diets containing GH diets remains uncertain, but may be partly associated with the higher feed intake previously reported for this diet [19] or the preservation method of the forage (i.e., hay *vs.* silage). It has been reported that ALA content in tissues is often higher, when hay as opposed to silage is fed due to a higher efficiency of transfer from the rumen to the tissue [40]. This could be related to changes in forage lipids, during preservation and/or ruminal lipid metabolism [41]. The process of ensiling has been shown to increase the levels of PUFA that are BH in the rumen due to lipolysis in the silo, which occurs as a result of increased moisture content during fermentation [42]. Biohydrogenation of PUFA's in the rumen has been shown to be lower in hay compared to ensiled forages, and therefore with hay a greater percentage of PUFA bypass the rumen and are available for uptake in the duodenum [40], [43]. This may be a result of differences in physical properties of the forage impacting microbial attachment [43] or shifts in microbial communities that result in differences in lipid metabolism and outflow of FA [44]. In this investigation, the mean percentage concentration of ALA in subcutaneous fat (0.44–0.50% of total FA) of steers fed diets containing FS was higher than the mean percentage concentration of ALA in the subcutaneous fat of animals fed FS in high-grain diets (0.09–0.12%, [33]), but lower than those found, when feeding FS in 50∶50 forage:grain (0.73–0.79%, [7]) or high-forage diets (0.8 7%, [13]; 1.1%, [9]). However, the differences in ALA across these trials could be related to a number of factors within these experiments including feeding management, forage type and quality or animal variability.

The finding that CLNA proportions in the subcutaneous fat were accentuated by feeding diets containing FS, as opposed to feeding SS, was anticipated as ALA is known to initially get isomerised to CLNA as it undergoes BH in the rumen [45]. Current findings agree with our earlier results, when feeding GH or barley silage diets containing FS [7] and FS combined with RC [10]. Previous cell culture and animal model studies have demonstrated that plant-derived CLNA can improve biomarkers of cardiovascular health and has anti-inflammatory, immune-modulatory, anti-obesity and anti-carcinogenic properties [46]. Therefore, addition of FS to high-forage diets to achieve greater levels of CLNA in beef fat may be perceived as a positive outcome from a human health perspective.

The increase in AD isomers largely derived from ALA, including *t*11,*c*15-18:2 the major AD isomer in steers fed diets containing FS was expected as ALA is isomerised to CLNA, before BH [45]. These findings agree with previous reports with diets containing FS [7], [10], [47]. Some AD isomers (*t*8,*c*12-; *c*9,*t*12-; *c*9,*c*15-18:2) reported by Mapiye et al. [10] and Nassu et al. [7] were increased by FS, but in the present study these isomers were preferentially enriched by diets containing SS as reported by Bessa et al. [47]. This could imply that these isomers are found in common with BH pathways for both LA and ALA. The inconsistencies in AD proportions seen between steers fed GH *vs.* RC, may also reflect changes in the physical state of the RC as a result of ensiling increasing the degree of BH of PUFAs in the rumen [40], [41], as enhanced microbial attachment to silage may have decreased the rumen bypass of PUFAs [43]. The observation that the proportions of AD likely largely derived from ALA were higher, when feeding diets containing FS as opposed to SS supports previous observations by Jenkins et al. [48] and Doreau et al. [49]. It was found that BH of ALA proceeds mostly through pathways incorporating AD *vs.* CLA, whereas BH of LA proceeds mostly through CLA. Definitive experiments on the impact of AD isomers on human health are required before recommendations can be made about including SS or FS in ruminant diets.

As found previously in ruminants [13], [47], [50], supplementing high-forage diets with SS as opposed to FS enhanced the proportions of CLA isomers with the first double bond from carbon 7 to 10 and *t*-18:1 isomers with double bonds between carbons 6 and 12. This is because of the common precursor LA, which is abundant in SS containing diets. Feeding cattle, a blend of oils or oilseeds rich in ALA and LA has previously been shown to result in a synergistic accumulation of VA [51] and CLA in the tissue [52]. Therefore, further investigations regarding whether blending SS and FS in high-forage diets would simultaneously enhance concentrations of VA, RA and *n*-3 PUFA in beef tissues are necessary. Furthermore, the blending of ALA and LA with long chain PUFA such as EPA and DHA, which are known inhibitors of BH of 18:1 to 18:0 in the rumen [53], [54] merits investigation.

In this investigation, proportions of RA and VA in subcutaneous fat (2.1% and 2.6%, respectively) of steers fed diets containing SS were greater than those previously reported, when high-forage diets were supplemented with oils or oilseeds [7], [55], [56]. Conversely, mean percentage concentrations of RA and VA in subcutaneous fat was determined to be less than that observed, when grass pastures were grazed by cattle supplemented with FS or sunflower oil (2.7–3.9% and 7.5–9.7%, respectively; [13], [56]) or RC with FS (2.9% and 5.9%; [10]). Variation in mean percentage concentrations of RA and VA across studies could be due to the effects of non-oil components of the diet on rumen BH. In the current study, the sunflower seeds were relatively low in oil (29.5%) compared to normal values of ≈l 40% [57]. As a result, the amount of SS that had to be added to the diet to get 5.4% oil was relatively high (18.4%), necessitating the addition of hulls that have a low energy value. Consequently, to balance the digestible energy content across diets, straw had to be added to flaxseed diets, leading to a diminished overall quality of forage in these diets. Therefore, the present findings strongly emphasize the importance of the non-oil components of the diet when trying to enrich for BH intermediates, particularly RA and VA, in beef. Overall, direct comparisons among studies are often difficult due to RA commonly being combined with other CLA isomers such as *t*7,*c*9-18:2, and VA containing more than one *t*-18:1 isomer.

The observation that feeding diets containing SS as compared to FS increased *t*10-18:1 confirms earlier reports by AbuGhazaleh and Jacobson [58] and Martínez Marín [59] that the BH of ALA as opposed to LA is less prone to promote *t*10-18:1 formation. The relative amounts of *t*10- and *t*13/*t*14-18:1 in the current study, are lower than those found by Juárez, et al., [8], whereas the relative amounts of *t*15- and *t*16-18:1 are less than those obtained by Nassu, et al., [7] and Mapiye et al [10]. These inconsistences could be attributed to differences in time on feed or levels, types and forms of forage fed in these experiments.

The increase in the proportions of minor CLA and *t*-18:1 isomers observed when feeding GH as opposed to RC may be partly associated with changes in the composition of ruminal microbes [60] or differences in ruminal and duodenal flow rates between these two forages [61]. For CLA and *t*-18:1 isomers, effects of oilseed type on these FA were greater than that of forage type.

This experiment showed that measured concentrations of *c*-MUFA isomers in subcutaneous fat were decreased when feeding SS as opposed to FS, which concurs with previous findings [62], [63] when feeding LA- and ALA-rich diets to dairy cows and goats, respectively. Overall, the levels of *c*-MUFA in subcutaneous fat when feeding PUFA are indicative of their dietary levels, the level of PUFA BH to *c*-MUFA in the rumen and the capacity of PUFA/or its BH products to inhibit stearoyl-CoA desaturase (SCD) activity, which converts 18:0 to *c*9-18:1 (oleic acid) in subcutaneous fat [64]. This further suggests that either LA has a slower BH rate and extent of ruminal BH in the rumen than ALA [36] or is more effective in down-regulating SCD activity than ALA [62]. In the present study, reductions in the proportions of total and individual *c*-MUFA isomers in steers fed SS diets could also relate to displacement of *c*-MUFA due to increased formation of *t*-18:1 during BH of PUFA in the rumen, as reflected by the elevated levels of total and individual *t*-18:1 isomers in subcutaneous fat.

Steers fed diets containing FS had a greater percentage of total BCFA as well as individual BCFA and odd-chain FA in subcutaneous fat than steers fed SS. This suggests that ALA did not inhibit rumen microbial FA synthesis as much as LA. In general, the decrease in BCFA and odd-chain FA in ruminant products is related to a direct inhibition of PUFA on microbial FA synthesis [65]. Given that BCFA have the potential to reduce necrotizing enterocolitis [66] and cancer [67], [68] in humans, diets that increase levels of BCFA in beef are desirable from a human health perspective.

### Rumen bacterial profiles

The fatty acid results of the present experiment demonstrate that feeding oilseeds in combination with diets containing a greater proportion of forage directly effects the mean percentage concentration of PUFA and their BH products in beef fat. However, there are many factors that simultaneously affect the amount and variation of PUFA and their BH products, making ad hoc estimation of fatty acid profiles extremely challenging. In order to perform such calculations a multifactorial approach is required, and among the potential variables, the bacterial ecosystem of the rumen will undoubtedly have an overriding influence. Where historically others have been able to culture isolated bacterial species which metabolize dietary FA [53], [69], the global perspective of how the entire population may be changing in response to diet and correlate with differences in FA profiles is lacking. This prompted our study taking advantage of current technologies to more fully characterize differences in rumen bacterial populations and their relationship to diet and FA deposited in beef. Previous research has cultured individual bacteria to examine their role in BH [69], [70]. However, the sensitivity of rumen bacteria to temperature, pH, osmolality, and the bacterial competition, quorum sensing, nutrient limitations and syntrophism within the rumen, makes it difficult to replicate this ecosystem in the laboratory [71], [72]. The use of pyrosequencing of the rumen ecosystem creates the opportunity to gain an overview of rumen bacterial populations simultaneously at any one point in time and compare it to other points in time. By looking at changes in the overall ecosystem within a variety of diets and animal hosts, one may be able to predict which bacterial populations are involved in rumen BH. This approach has been previously used to document the role of rumen bacteria in acidosis [73]. Although the ability of pyrosequencing to identify individual species is limited, the data generated could be used as a guideline for further research and when combined with culture techniques provide insight into the manipulation of individual rumen species involved in BH.

While overall sequence data was unable to fully describe the rumen bacterial ecosystem in cattle fed each diet due to limited coverage (Fig. S1), it was able to show that there were a large number of OTU's shared regardless of forage or oilseed type (Fig. 3). Therefore, despite dietary differences, there was a core group of microbes that was similar among all diets. However, changes in both ingredient and nutrient composition can impact the prevalence and density of specific groups or species without impacting the overall bacterial community structure [74]. While a number of genera were clearly impacted by diet (Table 3), variations in diet quality (*i.e.*, ADF, NDF) and composition (*i.e.*, inclusion of barley straw *vs.* barley grain) confounded the impact of oilseed or forage source on bacterial abundance. Regardless of diet, more than half the genera in Table 3 were members of the phylum Firmicutes, class Clostridia, order Clostridiales. This order is known to be Gram-negative, obligate anaerobes with a large representation in the rumen ecosystem [75]. However, despite the extensive diversity of this genus, previous research has indicated a possible link between some of these species and rumen BH [76].

When the 25 bacterial genera impacted by diet where compared in a correlation analysis to individual and groups of FA, 19 showed a significant correlation to one or more of the FA's (Table 4). Of these 19, five genera including *Acidaminobacter*, *Clostrium IV*, *Fibrobacter*, *Marvinbryantia* and *Olsenella* showed correlation to 4 or more individual FA's. *Clostridium IV* were highly negatively correlated to levels of CLNAisomers *c*9,*t*11,*t*15-18:3 and *c*9,*t*11,*c*15-18:3, as well as ALA. Increased levels of ALA and its long chain derivatives were found in the subcutaneous fat of cattle fed diets containing FS. Therefore, the increased percent abundance of *Clostridium IV* and *Ethanoligenens* in diets containing SS and not FS indicates that these two genera may be negatively associated with ALA and other n-3 PUFA in the rumen. However, several other genera including *Barnesiella*, *Butyrivibrio*, *Eubacterium* and *Olsenella* all showed increased abundance in diets with increased levels of at least one *n*-3 PUFA. Since ALA is considered a beneficial FA for human consumption [3]–[6], further research into the role of these organisms in rumen BH of ALA could elucidate how to increase ruminal bypass of ALA to adipose tissue.

*Anaerostipes* was the only genus that was found to be negatively correlated (*P*<0.001) to *n*-6 PUFA, which is known to inhibit BH in the rumen. Interestingly, *n*-6 PUFA were found to be highest in diets containing SS and *Anaerostipes* was also most abundant in RC-FS diets. This bacterial group may be also be involved in the rumen BH process and could be an indicator of inhibition of BH.

Post hoc analysis comparing cattle with high *vs.* low levels of VA in subcutaneous fat showed a significant separation of means (*P*<0.001; Table 5). Individuals with the lowest levels of VA in subcutaneous fat were found to have rumen populations with a greater abundance of *Anaerophaga*, *Fibrobacter*, *Guggenheimella*, *Paludibacter* and *Pseudozobellia*. Increasing amounts of VA in adipose tissue has been a target for ongoing research as it is a precursor for RA and may have beneficial health effects on its own [77]. However, some rumen bacteria are able to hydrogenate VA to steric acid [48], [69]. Therefore, bacteria found at significantly increased levels in cattle with low as compared to high VA in tissue may have greater VA BH activity in the rumen.

The main bacterial species within the rumen which is known to impact FA metabolism is *Butyrivibrio proteoclasticus* [69]. Similar to previous research, a relation between FA metabolism and *Butyrivibrio* was noted in the current experiment. However, increased levels of *Butyrivibrio* were related to increased levels of *n*-3 PUFA in the tissue and not increased levels of SFA as has been previously noted [69]. However, the current study was unable to identify changes in percent abundance of bacterial groups at a species level and therefore other *Butyrivibrio* spp may also be involved in rumen BH. Previous research has also indicated that *Propionibacteria acnes*, *Selenomonas ruminantium*, *Enterococcus faecium*, *Staphylococcus sp.*, *Flavobacterium sp.* and *Streptococcus bovis* may also play a role in the hydrogenation of *trans* FA to SFA [69]. While none of these bacterial groups were noted as being significantly different in this study, a number of previously unreported bacterial genera were identified as being correlated, either positively or negatively, to the levels of various FA in subcutaneous fat. These additional bacterial genera may also play a role in ruminal BH that has not yet been elucidated using traditional microbial culturing techniques. Further research is required to clarify the role of not only these bacterial groups but also of ciliate protozoa in relation to dietary changes impacting the FA profiles of adipose tissue in ruminants.

## Conclusions

The present study showed that cattle fed high-forage diets supplemented with SS had increased proportions of VA, RA and LA in subcutaneous fat, whereas supplementing with FS increased *n*-3 PUFA, CLNA, and AD. Present results demonstrate that dietary changes have the potential to produce beef with enhanced levels of PUFA BH products. However, in order to produce beef with consistently increased levels of PUFAs, further knowledge regarding how the type of PUFA, composition of the non-oil fraction of the diet and the type and level of feed processing impact PUFA deposition in the subcutaneous fat is required. Optimizing ruminal outflows of BH products will also require a better understanding of the precise role of rumen microbes and how the individual animal's genetics contribute to inter-animal variation. While some bacterial genera in this experiment were found to be correlated to specific FAs, the effect of diet on lipolytic rumen microrganisms and preferential pathways for BH requires greater insight by combining molecular techniques with classical culture techniques. Bacterial genera positively correlated to increasing BH intermediates in the present experiment need to be further researched in order to confirm whether these correlations are consistent over a range of diet types.

## Acknowledgments

Special thanks are extended to staff at the Lacombe Research Centre (LRC) Beef Unit of AAFC for animal care, animal management and sample collection. The slaughter and processing of the cattle by the LRC abattoir staff is gratefully acknowledged. Contributions of the meat grading and quality staff at the LRC to the result are appreciated. Ms. I.L. Larsen and Dr. D. Petri are acknowledged for their valuable assistance in statistical analysis.

## Supporting Information

Figure S1 **Rarefaction curves representing estimated coverage of bacterial sequences representing unique taxon for each of the diets (GH-SS: hay-sunflower seed, GH-FS: hay-flax seed, RC-SS: red clover-sunflower seed, RC-FS: red clover-flax seed).**
(DOCX)
